# Phage Cocktail Designed for Wastewater Bioremediation Has Limited Effect on Crustacean Filtrator Microbiome Diversity and Health

**DOI:** 10.1111/1758-2229.70329

**Published:** 2026-04-08

**Authors:** Marta Grabska, Adrian Gorecki, Hannah V. Pye, Evelien M. Adriaenssens, Malgorzata Grzesiuk

**Affiliations:** ^1^ Department of Biochemistry and Microbiology Institute of Biology, Warsaw University of Life Sciences (WULS) Warsaw Poland; ^2^ Quadram Institute Bioscience, Norwich Research Park Norwich UK; ^3^ Centre for Microbial Interactions Norwich Research Park Norwich UK

**Keywords:** *Daphnia magna*, ecotoxicology, host‐associated microbiome, life‐history parameters, phages

## Abstract

In this study we investigated the impact of a phage cocktail on *Daphnia magna* microbiome and the life‐history parameters. A mixture of four phages able to infect strains of *Klebsiella pneumoniae* , *Enterobacter* sp. and *Pseudomonas aeruginosa* was tested on three *D. magna* clones*.* The host‐associated microbiome composition in both the examined variants and the control was analysed using 16S rRNA amplicon sequencing. Additionally, the survival, growth rate, age, size at the first reproduction, and neonate per female were assessed. The analysis revealed minor shifts in microbial composition following phage exposure. Nevertheless, results showed that the phage cocktail increased microbiome diversity. None of the life‐history parameters studied were affected by the presence of the phage cocktail, and no adverse effects were observed. The results indicated that under laboratory conditions the phage cocktail is safe for *D. magna* and its microbiome.

## Graphical abstract

Phage cocktail designed for wastewater bioremediation is safe for freshwater filtrators. *D. magna* cultured with phages showed no significant changes in life‐history parameters and minor shifts in the microbial composition with increase in alpha diversity.

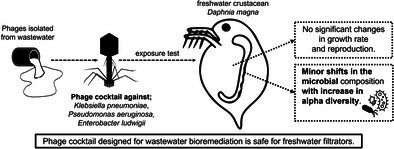

## 1 Introduction

Wastewater purification is essential for protecting water resources, public health, and the environment (Hanjra et al. 2012). Well‐established purification processes in wastewater treatment plants (WWTPs) facilitate the removal of a wide range of contaminants like solid waste, harmful pathogenic bacteria, viruses and toxic chemicals, for example, aromatic compounds or heavy metals, before they are discharged back into water bodies (Sims and Kasprzyk‐Hordern 2020; Elsaid et al. 2021; Qasem et al. 2021). Using appropriate purification methods prevents waterborne diseases from spreading, protects aquatic ecosystems from contamination, and supports the potential for water recycling to be used in agriculture, industry or as drinking water. Reducing the release of inadequately treated wastewater into freshwater and marine ecosystems helps prevent the accumulation of nutrients, heavy metals, pharmaceuticals, and other biological and chemical contaminants that can disrupt water quality and aquatic life. By limiting these pollution sources, aquatic biodiversity and ecosystem function are protected, while sustainable use of water resources for human and environmental needs is supported (Obaideen et al. 2022).

Antimicrobial resistance (AMR) has recently been prioritised by the World Health Organization (WHO) as one of the top 10 global public health threats facing humanity (WHO 2021). Unfortunately, the number of reports highlighting the presence of AMR in wastewater has increased (Manaia et al. 2018). Untreated sewage often contains a mix of human waste, pharmaceuticals including antibiotics, industrial chemicals and microorganisms from hospitals, farms and households (Rizzo et al. 2013; Mao et al. 2015; Wang et al. 2020). This leads to the selection of microbial opportunists, including multidrug‐resistant bacteria that can survive exposure to many different antibiotics and harsh environmental conditions (Tello et al. 2012). These bacteria can transfer their resistance to other microbes via horizontal gene transfer (HTG) in the receiving environment and animals, exacerbating the global AMR crisis (Liu, Thomsen, et al. 2022; Michaelis and Grohmann 2023). Therefore, novel methods for sewage treatment optimisation are essential to target and eliminate resistant organisms and reduce the impact of AMR on public health. Special attention should be given to the ESKAPEE group of bacteria ( *Enterococcus faecium* , *Staphylococcus aureus* , *Klebsiella pneumoniae* , *Acinetobacter baumannii* , *Pseudomonas aeruginosa* , *Enterobacter* spp. and *Escherichia coli* ), as they are responsible for the highest number of deaths related to bacterial infections. These pathogens exhibit high antibiotic resistance, making treatment significantly more challenging and increasing the risk of fatal complications, especially among hospitalised patients and those with weakened immune systems (Miller and Arias 2024).

Bacteriophages (phages) can be used as an alternative to antimicrobials to eradicate AMR bacteria, offering a natural and targeted approach to reduce this growing threat (Kulshrestha et al. 2024). Phages are viruses that specifically infect and kill bacteria. Unlike broad‐spectrum antibiotics, phages can be tailored to target specific strains of AMR bacteria without harming beneficial microbes. In wastewater treatment, phages could be introduced to selectively eliminate resistant bacteria before the wastewater is released into the environment, preventing the spread of resistance genes (Runa et al. 2021). However, because phages are biologically active agents, their use in sewage treatment requires extensive testing, such as ecotoxicological assessments, to ensure they are safe for the environment and ecosystems (Tkaczyk et al. 2021). Phages interact with bacterial populations in complex ways, and while they specifically target bacteria, their long‐term ecological impacts must be carefully studied in different animal models inhabiting the receiving environment.

*Daphnia* sp. are common freshwater crustaceans that play a central role in freshwater food webs as primary consumers, linking phytoplankton and microbial producers to higher trophic levels. By grazing on algae, bacteria, and detrital particles, they regulate primary production and influence the structure of planktonic communities. In ecotoxicological research, daphnids are widely used as bioindicators due to their high sensitivity to a range of chemical compounds. With their short life cycle, parthenogenetic reproduction, and transparent carapace, *Daphnia* sp. are recognised as a model organism for evaluating both acute and chronic toxicity in toxicological studies (Ebert 2022).

In this study, the impact of a phage cocktail on *Daphnia magna* was tested. Three *D. magna* clones were cultured with a phage cocktail comprised of four phages targeting a selection of multidrug‐resistant ESKAPEE pathogens: *K. pneumoniae* , *P. aeruginosa* and *Enterobacter* sp. Life‐history parameters and the microbiome were analysed to determine the effect of the phage cocktail on the freshwater filter feeder *D. magna* . Life‐history parameters provide valuable insights into the condition of an organism, which in *Daphnia* sp. is closely linked to its microbiome. The microbiome plays a crucial role by supplying essential nutrients that influence growth, reproduction and resistance to stress factors (Akbar et al. 2022). Therefore, environmental factors that alter the microbiome, such as phages, may ultimately affect the host's condition. The aim of the presented study was to indicate whether a phage cocktail is safe for *D. magna* and its microbiome under controlled laboratory conditions. Analysing both life‐history parameters and the microbiome enables a more comprehensive evaluation of the impact of phage presence on the fitness of *D. magna* .

## 2 Experimental Procedures

### 2.1 *D. magna* Clones

Three *D. magna* clones, referred to as B, D and P, were used. The clones came from three reservoirs: (i) B clone isolated from Binnensee, Germany (53.23°N, 12.37°E), (ii) D isolated from Domin pond, Czech Republic (49.00°N, 14.43°E) and (iii) P originated from the city park ponds in Warsaw, Poland (52.12°N, 21.00°E).

Experimental monocultures were established by isolating a single female of each clone from the clone library of the Department of Biochemistry and Microbiology (Warsaw University of Life Sciences, Poland). Second‐clutch neonates (< 16 h old) from each subsequent generation were used for further culturing and experimental assays. To standardise the pre‐experimental conditions, animals of each clone were cultured for at least three generations in the laboratory before the experiment. Both pre‐experimental and experimental daphnids were cultured under constant conditions: in a climate cabinet (ST 1450 PS SMART) adjusted to 20°C ± 0.5°C, summer photoperiod (16L:8D), and fed daily with green algae *Acutodesmus obliquus* (SAG 22.81, from Göttingen, Germany) cultured in sterile Bold's Basal Medium (BBM) modified by Guillard (Bischoff and Bold 1963; Guillard 1975) at a non‐limiting growth concentration of 1 mg C_org_ L^−1^ (Lampert 1987). Animals were cultured in ADaM (Aachener Daphnien Medium) (Klüttgen et al. 1994).

### 2.2 Bacteriophage Isolation, Amplification and Purification

Four phages were isolated from wastewater using a traditional wastewater enrichment method (Van Twest and Kropinski 2009) against *K. pneumoniae* , *Enterobacter* sp. and *P. aeruginosa* . Phage 1 was isolated using *P. aeruginosa* strain PAO1 as the host. Phages 2 and 3 were both isolated using *K. pneumoniae* strain er9204 as the host, and Phage 4 was isolated using *Enterobacter ludwigii* strain HVP31 as the host. Briefly, an aliquot of mid‐log‐phase bacterial culture was mixed with filtered wastewater (0.45 μm pore size) in a 1:5 ratio and grown for 18 h at 37°C. The resulting supernatant from the phage enrichment was filtered (0.45 μm pore size), and a 10 μL aliquot of each enrichment was dispensed onto a confluent lawn of host bacterium prepared by mixing the host bacterium at approximately 1 × 10^8^ colony‐forming units per millilitre (CFU/mL) with soft agar (double‐agar overlay method), as described by Kropinski et al. (2009). The appearance of zones of clearing on the plates indicated the presence of a phage, and these were subsequently subjected to three rounds of purification to isolate single plaques, ensuring a single plaque morphology of consistent size and margin was observed for each phage, before being amplified in solid agar plates. Phage titre was determined using the double‐agar overlay method, and phage lysates were stored at 4°C in phage buffer (75 mM NaCl, 10 mM MgSO_4_, 10 mM Tris, pH 7.5, 0.1 mM CaCl_2_) until required.

Each phage stock was purified using anion‐exchange chromatography on an ÄKTA Pure (Cytiva) protein purification system, as outlined in Vandenheuvel et al. (2018). To generate large‐volume, high‐titre phage stocks of each phage, an initial optimisation was conducted at three different multiplicities of inoculation (MOIs), and the condition(s) at which the highest titre was achieved were selected for purification. Specifically, individual conical flasks containing 50 mL growth media were inoculated with a 50 μL aliquot of an overnight host culture at approximately 1 × 10^9^ CFU/mL and incubated for 3 h at 37°C with shaking set to 200 rpm. Flasks were inoculated with each phage at an MOI of 0.1, 0.01 and 0.001. Phage‐inoculated flasks were incubated for a further 6 h at 37°C with shaking at 200 rpm. The phage lysates at the MOI that produced the highest titre of each phage were pelleted by centrifugation at 4000 × *g* for 20 min, and the supernatant was filtered through a 0.45 μm and then a 0.22 μm PES filter. If there was no difference in phage titre for each MOI, then the flasks were combined and pelleted by centrifugation, as before. The filtered phage lysate was loaded onto a CIMmultus QA‐8 mL monolithic column (Sartorius BIA Separations) and collected in 5 mL fractions using an elution buffer of 40% 20 mM Tris–HCl (pH 7.5) and 2 M NaCl. Phage titre was determined using the double‐agar overlay method, and the most concentrated purified phage fractions were combined and stored at 4°C until required.

A 5 mL aliquot of Phages 1–3, and a 10 mL aliquot of Phage 4 were shipped from the Quadram Institute Bioscience (Norwich, UK) to the Department of Biochemistry and Microbiology at the Warsaw University of Life Sciences (Warsaw, Poland) for use in *D. magna* experiments. The phage samples were transported in a well‐insulated shipping container equipped with cooling packs to maintain a low and stable temperature during transit. Upon arrival and unpacking at the receiving laboratory, the internal temperature of the shipment was measured and confirmed to be approximately 4°C, indicating appropriate cold‐chain conditions throughout transport. Phages were diluted using ADaM culture media to approximately 1 × 10^8^ plaque‐forming units per millilitre (PFU/mL), and 4 mL of each diluted stock was combined. The resulting phage cocktail had a volume of 16 mL at ~1 × 10^8^ PFU/mL.

### 2.3 Experimental Setup

The experimental animals were juveniles from the second clutch of the second generation, all descended from a single founding female. The 20 juveniles were randomly assigned to treatment and control groups, ensuring full randomisation, and all were time‐synchronised by hatching within a 16‐h window. Life‐history parameters were assessed using about 10 individuals per treatment from each of three clones, with the clones serving as independent biological replicates. Daphnids were kept together in 1 L vol. at a density of one individual per 100 mL of medium. All three clones were tested simultaneously. Additionally, at the end of the experiment, all animals were collected for microbiome analysis.

Experimental media were as follows: (i) ADaM—control, (ii) phage treatment—four phages in equal proportions at a final concentration of 10^5^ PFU/mL in ADaM. Because no known bacterial hosts for the tested phages were expected to be present, and the phages have a narrow host range, replication within the medium was unlikely. Therefore, the culture medium was renewed every second day, and *Daphnia* received a fresh dose of phages at the same concentration to maintain stable phage levels. Under these conditions, phage persistence was assumed to be influenced only by environmental factors (Ranveer et al. 2024; Williams et al. 2024), which were kept constant throughout the experiment. Other experimental conditions were the same as in pre‐experimental culturing.

### 2.4 *D. magna* Microbiome Analysis

For microbiome analysis, experimental animals were sampled after producing their first clutch of offspring. Total DNA was extracted from 54 to 138 mg of *D. magna* per sample using a Fast DNA Spin Kit for Feces following the manufacturer's guidelines. The bacterial *16S rRNA* genes were amplified from the extracted total DNA using the primers Bac341F (Muyzer et al. 1993) and Bac805R (Herlemann et al. 2011). Initial raw data quality control was checked using FastQC (version 0.12.1) (Andrews 2010). Sequences were imported into the QIIME2 software package (release 2021.11) for subsequent analysis (Bolyen et al. 2019). Read sequences were truncated (281/242 forward/reverse), and quality filtering, denoising, paired‐end reads merging, and de novo chimaeras removal were performed using the DADA2 plugin in order to obtain amplicon sequence variants (ASVs) (Callahan et al. 2016). Bacterial taxonomy was assigned for each of the ASVs using a pre‐trained Naive Bayes classifier based on the Silva 138 SSU database (Quast et al. 2012). Raw sequencing data are available at the European Nucleotide Archive (ENA) under BioProject accession number PRJNA1272056 and sample accession numbers SRX29048801–SRX29048806.

### 2.5 *D. magna* Life‐History Parameters

Survival rate was monitored during the whole experiment. For each treatment and clone, 4‐day‐old daphnids (*n* = 5) were photographed and measured in order to calculate their individual juvenile somatic growth rate (Lampert and Trubetskova 1996). All individuals from each clone were further observed to determine the age (AFR) and size (SFR) at first reproduction—defined as the day when eggs first appeared in the brood chamber—and number of neonates per female (*n* = 8**–**10). The length was obtained by photographing the *D. magna* individuals using an NE620 Nexcope microscope with a DLT Cam Pro 8.3 M camera. The animals were measured using DLTCamViewer software (exact to within 0.01 μm). Length increase was converted to juvenile somatic growth rates (g_i_) according to the formula *g*_*i*_ = (ln[*L*_*t*1_] − ln[*L*_*t*0_]) / Δ*t* where (*L*_*t*0_)—length of animals at the beginning of the experiment < 12 h old, *L*_*t*1_ 4‐day‐old daphnid, Δ*t*—time (in days) between the beginning of the experiment and the point of the animal photographed. Body length was measured as the distance between the upper margin of the eye and the posterior edge of the carapace at the base of the tail spine; the selected measurement points are illustrated in Figure S1. The sample sizes for each individual used in the analyses are provided in Table S1.

### 2.6 Statistical Analysis

We used a two‐way ANOVA followed by a Tukey post hoc (at *α* = 0.05) to test the effect of treatment on the *D. magna* life‐history parameters (growth rate, age and size at the first reproduction, number of neonates per female). Analyses were performed using Statistix 10.0. Alpha diversity metrics for microbiome, we calculated using the scikit‐bio package in Python. Statistical analysis of microbiome data was conducted using the scipy and statannotations packages in Python (version 3.13.1).

Beta diversity was assessed using Bray–Curtis dissimilarities calculated from arcsine square root–transformed relative abundances. The effects of Clone and Treatment on microbial community structure were tested using permutational multivariate analysis of variance (PERMANOVA) with the adonis2 function in the R package vegan (R version 4.5.2) with 999 permutations and marginal tests for each factor. Single‐factor PERMANOVA models were also performed; for clone, the low number of replicates (*n* = 2 per clone) limited the number of possible permutations (complete enumeration, *n* = 719), and results were interpreted cautiously. Principal coordinate analysis (PCoA) was used to visualise beta diversity patterns.

## 3 Results

### 3.1 Phage Stock Preparation

The four phages included in the cocktail were originally isolated from wastewater against strains of *K. pneumoniae* , *Enterobacter ludwigii* and *P. aeruginosa* . Differing MOIs were used for technical optimisation for initial phage stock production. After purification using anion‐exchange chromatography, the titre of each phage remained similar to what was originally used as the input. Thus, each phage was further diluted to approximately 1 × 10^8^ PFU/mL, and then 4 mL of each diluted phage stock was combined to form the phage cocktail for use in *D. magna* experiments.

### 3.2 *D. magna* Microbiome

A total of 1,203,234 paired‐end reads were obtained from the DNA sequencing run. The mean read count per sample was 200,539 reads (min. 141,723, max. 234,430). During quality control, low‐quality reads and nucleotides were removed, resulting in a mean read count of 151,207 (min. 109,259, max. 177,478).

**FIGURE 1:**
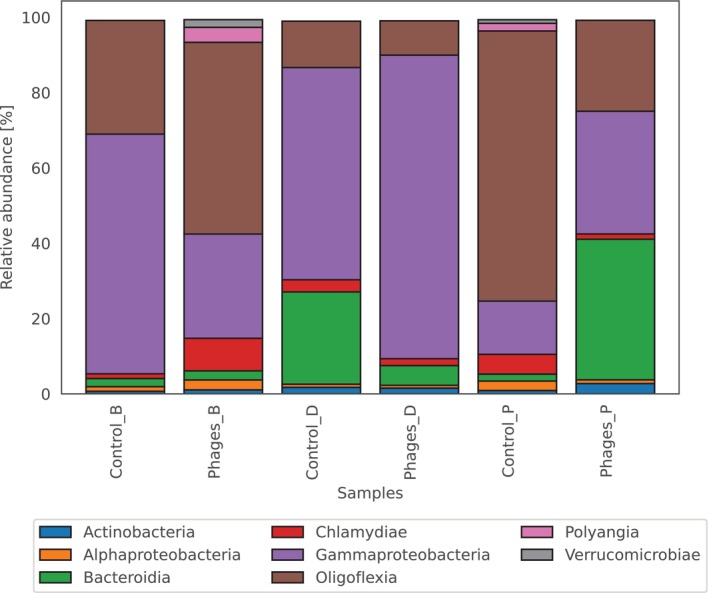
Relative abundance of bacteria classes in different samples. Only abundant classes are shown in this figure; classes in low relative abundances (< 0.5% in the sample) were filtered out from the plot.

The dataset presents the relative abundances of bacterial classes across six samples: (i) Clone B *D. magna* cultured in control conditions (Control_B), (ii) Clone D *D. magna* cultured in control conditions (Control_D), (iii) Clone P *D. magna* cultured in control conditions (Control_P), (iv) Clone B *D. magna* cultured with phage cocktail (Phages_B), (v) Clone D *D. magna* cultured with phage cocktail (Phages_D) and (vi) Clone P *D. magna* cultured with phage cocktail (Phages_P). The total proportion of bacterial types with an abundance not exceeding 0.5% ranged from 0.65% to 1.04% (Figure 1). Gammaproteobacteria were the most abundant class in Clone B, particularly in Control_B (63.66%) and Phages_B (80.62%), while Oligoflexia showed high representation in Control_D (50.91%) and Phages_D (71.74%). Bacteroidia was notably abundant in Phages_P (37.34%) and Control_P (24.50%), while Chlamydiae exhibited variability, with the highest levels in Control_D (8.66%) and Phages_D (5.25%). Polyangia and Verrucomicrobiae abundances were generally low, detected only in Control_D and Phages_D samples. These findings highlighted the diversity and specificity of bacterial community structures across different samples, with certain classes like Gammaproteobacteria and Oligoflexia being dominant in specific environments and clones, while others, such as Bacteroidia and Chlamydiae, display more variability in their prevalence and abundance.

**FIGURE 2:**
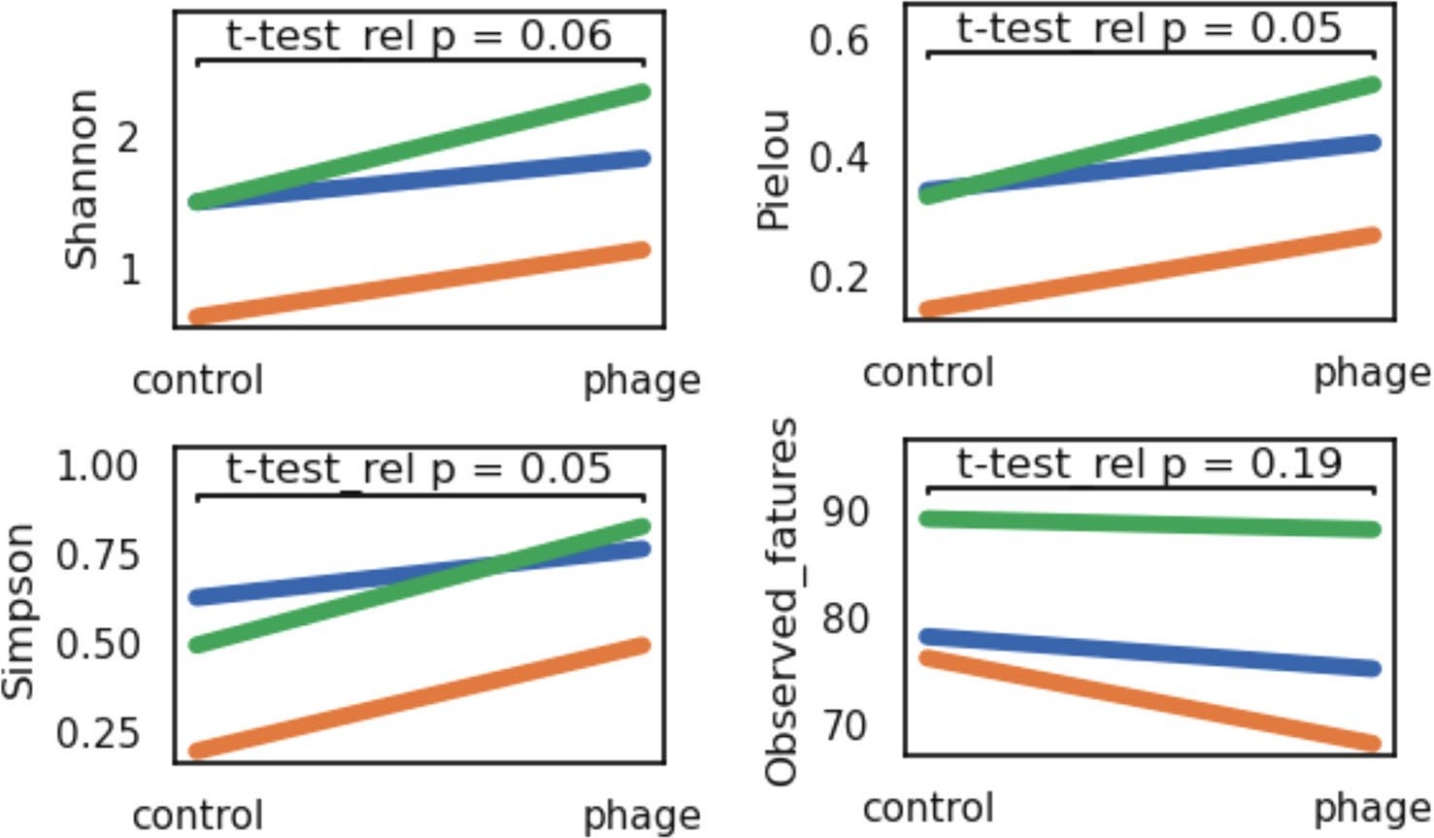
A line chart showing changes in alpha diversity indices of the host‐associated microbiome of *Daphnia magna* clones grouped per treatment. Green line: Clone D microbiome, blue line: Clone P microbiome and orange line: Clone B microbiome. The statistical analysis was performed using a paired Student's *t*‐test.

Phage‐treated samples generally showed higher diversity across several metrics. For Shannon diversity, samples treated with phages had higher values (*p* = 0.0633, *t* = −3.784), and a similar trend was observed for Simpson diversity (*p* = 0.0514, *t* = −4.238). Pielou evenness diversity also increased in samples treated with phages (*p* = 0.0527, *t* = −4.182). However, observed features, representing species richness, did not differ significantly between the experimental variants (*p* = 0.1946, *t* = 1.922), indicating that while samples treated with phages tend to have greater microbiome diversity, the overall number of distinct bacteria remains comparable across the groups (Figure 2).

Beta diversity was assessed using Bray–Curtis dissimilarity matrices calculated from arcsine square root–transformed data, and the effects of clone and treatment on microbial community structure were tested using permutational multivariate analysis of variance (PERMANOVA; adonis2, 999 permutations). In the additive model with marginal tests, Clone had a significant effect on community composition (*F* = 6.00, *R* ^2^ = 0.734, *p* = 0.0167), explaining approximately 73% of the observed beta diversity, whereas Treatment (control vs. phage exposure) did not show a significant effect (*F* = 2.35, *R* ^2^ = 0.144, *p* = 0.15). Single‐factor analyses were consistent with these results, with Clone showing a pronounced but statistically marginal effect when tested alone (*F* = 4.14, *R* ^2^ = 0.734, *p* = 0.0667; complete permutation enumeration, *n* = 719), while Treatment alone did not result in significant differences in community structure (*F* = 0.67, *R* ^2^ = 0.144, *p* = 0.60). Bray–Curtis dissimilarities between control and phage‐exposed samples within the same clone ranged from 0.207 to 0.258 (Clone B = 0.258, Clone D = 0.217 and Clone P = 0.207), indicating a high, though not complete, similarity in microbiome composition between treatments. These results are visually supported by PCoA of Bray–Curtis dissimilarities (Figure S2), which shows clustering of samples primarily by clone rather than by treatment. Control and phage‐exposed samples from the same clone largely overlapped in ordination space, consistent with the lack of a significant treatment effect detected by PERMANOVA.

To assess the impact of phage exposure on bacterial communities, we calculated genus‐level log2‐fold changes in relative abundance between phage‐exposed and control samples for the three clones (P, B and D). The top 20 genera by abundance were analysed to focus on biologically meaningful taxa. The heatmap (Figure S3) reveals clone‐specific responses to phage exposure. For example, *Hydrogenophaga* and *Flavobacterium* increased in most clones, with *Hydrogenophaga* showing a particularly strong increase in Clones B (log2FC = 2.22) and D (log2FC = 2.46), and *Flavobacterium* increasing moderately across all clones (log2FC = 0.49–1.08). In contrast, *Oligoflexus* displayed divergent responses: it increased in Clones P (1.42) and D (1.21) but decreased in Clone B (−2.05). Similarly, *Rheinheimera* showed a marked increase in Clones B (3.03) and D (4.34) while strongly decreasing in Clone P (−1.99). Some genera, such as *Acinetobacter*, exhibited dramatic clone‐specific decreases (B: −9.64 and D: −1.47), highlighting the heterogeneity of the bacterial response.

**FIGURE 3:**
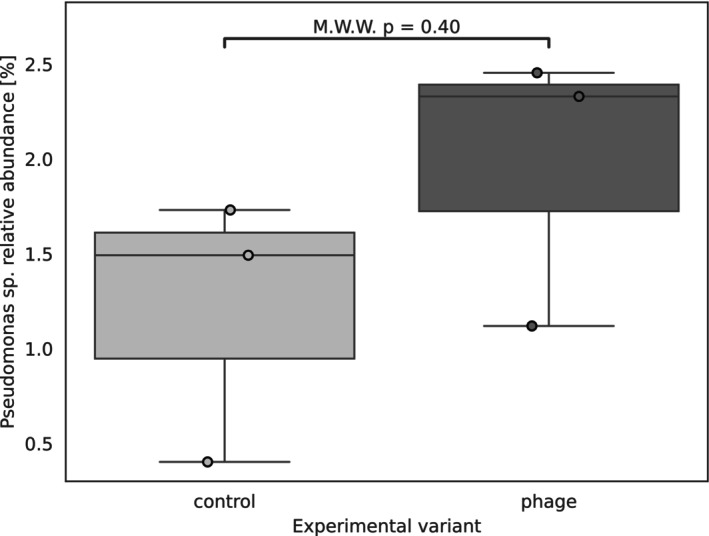
The distribution of *Pseudomonas* genus abundance in experimental variants. The boxplot shows the median (centre line), interquartile range (box), whiskers (1.5 × IQR), and outliers (dots outside the whiskers). Statistical analysis was performed using the Mann–Whitney–Wilcoxon test.

Overall, while some taxa responded consistently across clones, many showed clone‐specific shifts, suggesting that phage exposure can differentially affect bacterial composition depending on the host background. Because only three biological replicates were available (*n* = 3 clones), statistical tests were not performed, and these fold changes are presented exploratorily. Nonetheless, these results identify key genera that are responsive to phage exposure and may warrant further investigation. It is worth noting that among the three hosts of the applied phages, only representatives of the *Pseudomonas* genus were identified within the *Daphnia* microbiome, and with these data, no predictions could be made matching the phage to the potential host species. *Pseudomonas* was present in all analysed samples, with a relative abundance averaging 1.59% (ranging from a minimum of 0.41% [Control_B sample] to a maximum of 2.45% [Phage_B sample]). An increase in the abundance of the *Pseudomonas* genus was observed in the phage‐treated samples; however, this change was not found to be statistically significant (*p* = 0.40) (Figure 3).

### 3.3 *D. magna* Life‐History Parameters

**FIGURE 4:**
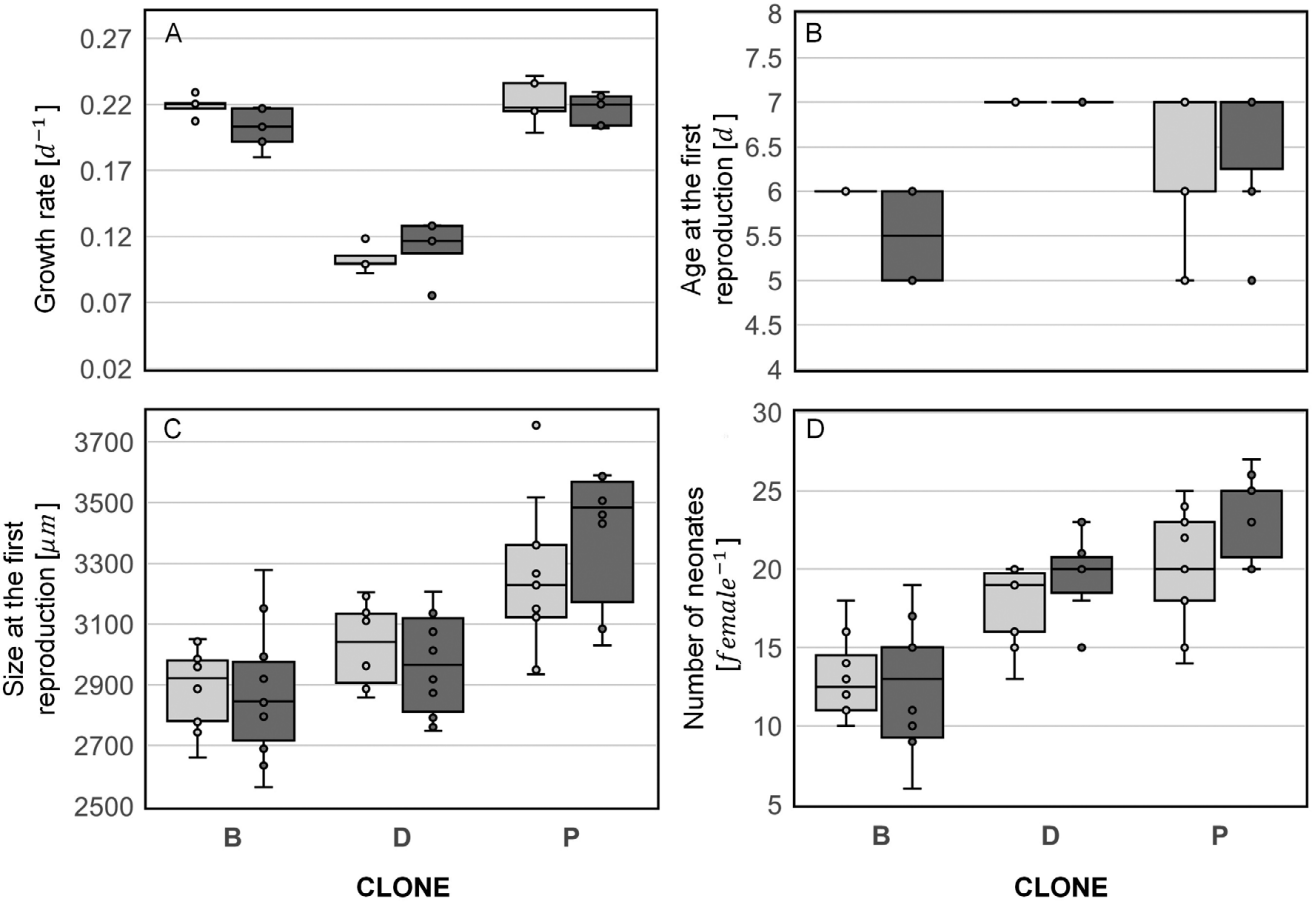
Comparison of life‐history parameters: growth rate (A), age (B) and size (C) at the first reproduction and number of offspring per female (D) across clones and treatment. The control group is represented by the light grey bar, while the phage‐exposed group is represented by the dark grey bar. Internal points are marked with circles. Outliers are plotted as individual points beyond the whiskers as circles.

Life‐history parameters (survival, growth rate, age and size at first reproduction and number of neonates per female) of *D. magna* were not dependent on culturing medium, but on the clone origin. No mortality was detected during the experiment. Within a single genotype, no significant difference in life‐history traits was observed. Exposure to the phage cocktail did not affect the overall condition of the tested animals (Figure 4 and Table S1). Growth rate for Clones B and P were non‐significantly lower for phage‐exposed animals (by 7.8% and 2.4%, respectively) relative to controls. However, phage‐exposed animals of Clone D had a higher (by 7.8%) growth rate than control animals, while the difference was also not significant. Clone B animals cultured with phages laid their first clutch into the brood chamber on average 0.5 days earlier than the control group, were about 0.5% smaller at the first reproduction and had fewer offspring (4% relative to the control), but the differences were not significant. For Clone D, phage‐exposed *D. magna* initiated their first reproduction at the same age as the control group (7 days) and at a slightly smaller size (2.2%). On average, females in the phage‐treated group produced 1.8 more neonates per clutch. None of these differences were significant. Animals of Clone P exposed to phages initiated first reproduction on average 0.2 days later than the control, were 4.1% larger, and had more offspring (an average of 3.7 more neonates per clutch). These differences were not statistically significant (Figure 4 and Table S1).

## 4 Discussion

Interest in using phage cocktails for wastewater bioremediation is increasing; however, the impact of phage on the flora and fauna associated with these environments is largely underexplored (reviewed in Hegarty (2025)). In this study, we investigated the effects of exposure to a phage cocktail on the microbiome and life‐history traits of three genotypes of *D. magna* . These results indicate that phage exposure led to minor changes in the internal structure of the microbiome, reflected by increased alpha diversity indices, while host life‐history parameters remained unaffected. Importantly, both microbiome beta diversity and host fitness parameters were mainly shaped by host genotype rather than phage treatment, highlighting the crucial role of host genetic background in microbiome assembly and functional stability.

### 4.1 Phages Reshape Microbiome Without Increasing Taxonomic Richness

In this study, phage exposure did not increase the number of observed microbial features but increased diversity (Shannon and Simpson indices) and evenness across all clones. This result indicates that the observed increase in alpha diversity was not driven by the introduction of new taxa but rather by a redistribution of relative abundances. These shifts were associated with a reduction in the dominance of previously abundant taxa, leading to niche release and subsequent occupation of these niches by subdominant and rarer bacteria (Cooper et al. 2021). This interpretation is explicitly supported by the fold‐change analysis of individual bacterial taxa. Dominant and commonly reported members of the *Daphnia* microbiome, such as *Limnohabitans*, *Acinetobacter* and *Phenylobacterium*, decreased in relative abundance following phage exposure, whereas several subdominant or opportunistic taxa (e.g., *Flavobacterium*, *Rheinheimera*, *Hydrogenophaga* and *Microbacteriaceae*) increased. Such shifts are consistent with an effect in which perturbations weaken competitive exclusion by dominant taxa, allowing other bacteria to expand without altering community membership (Shade et al. 2012). This pattern aligns with ecological theory and observations from aquatic microbial systems, where perturbations often increase evenness while leaving species richness unchanged (Allison and Martiny 2008).

Our findings are consistent with studies conducted on different experimental models, indicating that phage treatment does not disrupt the composition of the host‐associated microbiota of freshwater organisms (Dissanayake et al. 2019). A study performed by Fiedler et al. (2023) has found that phage therapy minimally affected the water microbiota from the fish rearing system by changing the composition of the bacterial community but not altering alpha diversity. Significant changes have been observed only in cases where the phage host was present in the microbiota of the analysed organism (Fiedler et al. 2023). In another study, phage administration has been observed to change the gut microbiota of fish, also in the absence of a bacterial host for phages. Despite the changes in the gut microbial community revealed by β‐diversity analysis, no adverse effects on fish health and growth were observed (Donati et al. 2022).

### 4.2 Indirect Effects of Phages on Microbial Community

Phages, due to their specificity, act on a narrow group of bacterial hosts. Accordingly, they are considered to have limited efficacy in shaping complex microbial communities, including the gut microbiome. However, numerous reports suggest that phage therapy can modify the microbiome even in the absence of a host for the phage (Fiedler et al. 2023). In the study conducted by Silva et al. (2016), it was observed that the bacterial community in the gastrointestinal tract of fish was more altered due to phage therapy when the specific host was absent compared to the situation when the host was present. The phages used in our study are specific to bacterial species that do not constitute the most abundant component of the natural microbiome of *D. magna* , although *P. aeruginosa* has been identified in the gut microbiota of *D. magna* alongside other *Pseudomonas* species (Cooper and Cressler 2020; Freese and Schink 2011). In our experiment, we identified representatives of *Pseudomonas* sp., but their proportion did not change significantly after phage treatment, suggesting that they are either non‐host *P. aeruginosa* or different species of this genus. In contrast, *K. pneumoniae* and *E. ludwigii* do not colonise the gastrointestinal tract of *D. magna* as a part of its microbiome (Freese and Schink 2011). The reported presence of these bacteria in the gut has been most often the result of *Daphnia* sp. feeding in contaminated water sources, meaning their appearance requires the presence of bacteria in the immediate environment of the water flea (Le Coadic et al. 2012; Tromas et al. 2025). Both *Pseudomonas* sp. and *E. ludwigii* are considered opportunistic bacteria, and for this reason, the dominance of Pseudomonadaceae in *D. magna* has been associated with a decrease in their growth and reproductive parameters (Siciliano et al. 2015; Akbar et al. 2020; Taylor et al. 2025).

The observed microbiome changes probably result from indirect effects of exposure to the phage cocktail. It is likely that phages influence interactions between bacterial and phage (phageome) populations naturally residing in the *D. magna* organism, leading to subtle changes in microbial community dynamics (Hsu et al. 2019). Certain reports suggest that some phages may directly modulate immune responses in mammals, thereby influencing the microbiome. However, this type of interaction is unexplored in *Daphnia* sp. (Federici et al. 2021). The observed increase in microbiome diversity in *Daphnia* may be partly driven by the breakdown of viral particles and the subsequent release of their genetic material into the surrounding environment. Certain bacteria within the microbiome are competent and can utilise this extracellular genetic material, both as a source of nucleotides and as a supply of essential elements such as phosphorus, nitrogen and carbon (Nielsen et al. 2007). By providing these additional resources, phage‐derived genetic material may alter nutrient availability and promote the coexistence of a broader range of microbial taxa, thereby contributing to the observed enhancement of microbial diversity (Hua et al. 2021).

### 4.3 Host Genotype as the Key Determinant of Microbiome Structure

Beta diversity analysis revealed a significant effect of host clone on microbiome composition, whereas no significant effect of phage exposure was detected, despite observed changes in alpha diversity. These results are consistent with previous studies, which showed that *Daphnia* sp. from different regions maintained their unique microbiomes even after many years of laboratory cultivation (Frankel‐Bricker et al. 2020). In addition, the microbiota tends to be stable within individual clones grown under identical conditions, suggesting a strong host‐genotype influence on microbiome composition (Freese and Schink 2011). It is worth noting that *Daphnia* cultured in a laboratory, in a homogeneous medium and fed a single species of algae, have a poorer microbiome than individuals living in the environment (Houwenhuyse et al. 2025; Gurung et al. 2025). Presumably, the impact of phages on the microbiome of organisms living in the natural environment may be less significant. A more diverse microbiome is more stable and resistant to changes, such as the emergence of phages (García‐García et al. 2019).

Variation in microbiome composition among hosts can arise from differences in host physiology and immune regulation, which, together with environmental factors, contribute to shaping the microbiome (Macke et al. 2017). Our results extend these observations by indicating that genotype‐dependent control persists even under microbiome perturbation, limiting how far and in which direction microbial communities can shift.

### 4.4 Life‐history traits and microbiome background

The observed changes in microbiome structure were not accompanied by significant changes in host condition. Survival, growth rate, timing of reproduction and fecundity did not differ significantly between phage‐exposed and control animals, and no mortality was observed. This suggests that the *Daphnia* microbiome is functionally robust, so changes in bacterial composition do not necessarily lead to reduced host fitness. The observed variability in life‐history parameters between different *D. magna* clones confirms the existence of genetically determined differences in adaptation to local environmental conditions (Lampert 1993). Each clone represents a distinct genotype whose ability to adapt life‐history traits may be the result of both local adaptation and the evolution of phenotypic plasticity (Dodson 1989; Yampolsky et al. 2014). The observed differences reflect the evolutionarily shaped ability of specific genotypes to respond to environmental conditions, while the presence of phages remained a non‐significant factor.

These clone‐specific life‐history patterns were consistent with differences in baseline microbiome diversity. Clone D exhibited the highest initial diversity, which was maintained under control conditions and coincided with faster growth and higher reproductive output following phage exposure, suggesting greater microbiome stability. In contrast, Clone B showed the lowest initial diversity and tended to exhibit slight declines in diversity indices together with reduced growth and reproduction. Clone P displayed intermediate diversity and a largely stable microbiome, consistent with its limited phenotypic response to phages. Although these trends were not statistically significant, they suggest a positive association between initial microbiome diversity, microbiome stability, and host growth and reproductive performance (Macke et al. 2017).

### 4.5 Ecological and Evolutionary Aspects

Phage therapy has been successfully used on freshwater animals, especially fish, to treat bacterial diseases (Liu, Han, et al. 2022). Numerous in vivo experiments conducted on aquatic animal species (e.g., fish, crustaceans and molluscs) confirm the high effectiveness of phage therapy in controlling pathogens and reducing mortality in infected animals (Schulz et al. 2022). For this reason, phage therapy is considered to be a safer alternative to antibiotic therapy, so it can be used on healthy animals to prevent a disease (Nakai et al. 1999). The 12‐day oral administration of phage mixture (FpV4 and FPSV‐D22) to rainbow trout fry had no negative effects on the growth and condition of the control (healthy) fish (Donati et al. 2021). However, it should be emphasised that predicting the effects of phages on aquatic organisms depends on a combination of many factors, for example, physicochemical parameters, phage concentration and time exposure (Le et al. 2018; Nakai et al. 1999). The results of the therapy depend primarily on the composition of the phage cocktail and their ability to reach the host. Knowledge regarding the rate and conditions of virus uptake, especially phages, from water by *Daphnia* sp. remains very limited. Some studies suggest that *D. magna* has the potential to uptake avian influenza virus (AIV) from the surrounding water (Abbas et al. 2012; Meixell et al. 2013). Another study showed that *D. pulex* does not accumulate ranavirus in its tissues but instead leads to its faster inactivation in water (Johnson and Brunner 2014). In the study conducted by Ismail et al. (2020) investigating the ability of *D. magna* to accumulate bacteriophage T4 and enterovirus E11, it was shown that the water fleas do not actively uptake these viruses directly from the water, nor do they have the ability to accumulate them. However, virus uptake occurred indirectly while foraging through the transfer of viruses from a prey ( *T. pyriformis* ) to a predator ( *D. magna* ).

As the phages applied in this study were newly isolated, the primary objective was to evaluate their effects under well‐controlled laboratory conditions, enabling the generation of clear baseline data in a system with stable and defined microbiological parameters. This approach represents a necessary first step towards assessing the ecological safety of phage‐based strategies targeting bacteria harmful to humans.

Existing studies provide strong proof‐of‐principle evidence that bacteriophages can selectively control problematic microorganisms in wastewater treatment systems, including bacteria responsible for activated sludge foaming and bulking, biofouling, and the presence of selected pathogens, without significantly disrupting the functional treatment microbiome (Liu et al. 2015; Runa et al. 2021). However, most investigations remain limited to laboratory‐ or pilot‐scale experiments conducted under controlled conditions (Mathieu et al. 2019). To improve ecological relevance and assess long‐term effects, future studies should incorporate longer exposure periods, multiple phage dosages, and microcosm or mesocosm systems that better reflect real wastewater environments (Ranveer et al. 2024). Even if efficacy at scale is confirmed, practical implementation faces additional challenges related to large‐scale phage production, which requires intensive bacterial cultivation, bioreactors, and complex downstream processing, including purification, endotoxin removal, and concentration, potentially generating significant brine and waste streams. Sustainable industrial deployment will therefore depend on effective waste management strategies and robust environmental safeguards (Mohammadi et al. 2025). These technical limitations are compounded by the absence of dedicated regulatory frameworks in the European Union for the environmental application of bacteriophages, which currently represents a major barrier to transitioning from pilot studies to full‐scale implementation.

In our study, the direction of changes in the microbial community was not associated with any negative consequences for the growth and fecundity of *D. magna* . It should be noted that this study has certain limitations, such as the use of a single phage concentration and the lack of qualitative and quantitative analysis of phages in the gut of *D. magna* .

## 5 Conclusion

Treatment with a phage cocktail led to detectable but relatively minor changes in the microbiome composition of *D. magna* at the tested concentration. These changes increased alpha diversity by reducing dominance and increasing evenness, without affecting taxonomic richness and were strongly genotype‐dependent.

Further analyses demonstrated that neither the phages themselves nor the subtle changes in the microbiome affected the life‐history parameters of *D. magna* . Thus, while there is an observable effect on microbial composition, it does not translate into adverse biological consequences. These findings support the conclusion that phage‐mediated modulation of the microbiome can occur without measurable negative consequences for host performance and is unlikely to pose a substantial risk for freshwater organisms under the conditions tested.

## Author Contributions

**Marta Grabska:** conceptualisation (supporting); methodology (equal); validation (equal); formal analysis (lead); investigation (lead); data curation (lead); visualisation (lead); writing – original draft (lead); writing – review and editing (lead). **Adrian Gorecki:** conceptualisation (supporting); methodology (equal); formal analysis (supporting); writing – review and editing (supporting). **Hannah V. Pye:** methodology (equal); visualisation (supporting); resources (supporting); writing – original draft (supporting); writing – review and editing (supporting). **Evelien M. Adriaenssens:** supervision (equal); resources (supporting); writing – review and editing (supporting). **Malgorzata Grzesiuk:** conceptualisation (lead); methodology (equal); validation (equal); investigation (supporting); resources (lead); writing – review and editing (supporting); supervision (equal); project administration (lead).

## Funding

This work was supported by National Science Centre (UMO‐2021/03/Y/NZ9/00141) under the framework of JPIAMR‐ACTION GA no. 963864, the Medical Research Council (MR/W031205/1) and the Biotechnology and Biological Sciences Research Council (BB/X011054/1, BB/X011011/1).

## Conflicts of Interest

The authors declare no conflicts of interest.

## Supporting information


**Figure S1:** Representative microscopy image of *D. magna* used for the measurement of body size at first reproduction (SFR).
**Table S1:** Two‐way ANOVA testing the effect of medium (i) ADaM—control, (ii) Phage treatment—four phages cocktail and genotype (B, D and P clone) of *D. magna* life history parameters: growth rate, age and size at the first reproduction and neonate number per female.
**Figure S2:** Principal coordinates analysis (PCoA) of Bray–Curtis dissimilarities among bacterial communities from *D. magna* microbiome. Each point represents a biological replicate. Points are coloured according to Clone and shaped according to Treatment (control vs. phage‐exposed).
**Figure S3:** Genus‐level log2 fold change in bacterial relative abundance in phage‐exposed versus control samples across three *D. magna* clones (P, B, D). Heatmap colours represent log2 fold change, with red indicating an increase and blue indicating a decrease in phage‐exposed samples relative to controls. Genera are sorted by the average log2 fold change across clones.

## Data Availability

The data that support the findings of this study are available on request from the corresponding author. The data are not publicly available due to privacy or ethical restrictions.
