# Supplementary material for: Phage Cocktail Designed for Wastewater Bioremediation Has Limited Effect on Crustacean Filtrator Microbiome Diversity and Health
Source: Environ Microbiol Rep. 2026 Apr 8;18(2):e70329. doi: 10.1111/1758-2229.70329 (PMC13058927; doi:10.1111/1758-2229.70329)
Supplement: Supplementary file 1 — Figure S1: Representative microscopy image of D. magna used for the measurement of body size at first reproduction (SFR). Table S1: Two‐way ANOVA testing the effect of medium (i) ADaM—control, (ii) Phage treatment—four phages cocktail and genotype (B, D and P clone) of D. magna life history parameters: growth rate, age and size at the first reproduction and neonate number per female. Figure S2: Principal coordinates analysis (PCoA) of Bray–Curtis dissimilarities among bacterial communities from D. magna microbiome. Each point represents a biological replicate. Points are coloured according to Clone and shaped according to Treatment (control vs. phage‐exposed). Figure S3: Genus‐level log2 fold change in bacterial relative abundance in phage‐exposed versus control samples across three D. magna clones (P, B, D). Heatmap colours represent log2 fold change, with red indicating an increase and blue indicating a decrease in phage‐exposed samples relative to controls. Genera are sorted by the average log2 fold change across clones. [file EMI4-18-e70329-s001.docx]

**Phage Cocktail Designed for Wastewater Bioremediation Has Limited Effect on Crustacean Filtrator Microbiome Diversity and Health**

Marta Grabska, Adrian Gorecki, Hannah V. Pye, Evelien M. Adriaenssens, Malgorzata Grzesiuk

**Abstract**

In this study we investigated the impact of a phage cocktail on *Daphnia magna* microbiome and the life history parameters. A mixture of four phages able to infect strains of *Klebsiella pneuumoniae*, *Enterobacter* sp. and *Pseudomonas aeruginosa* were tested on three *Daphnia magna* clones*.* The host-associated microbiome composition in both the examined variants and the control was analyzed using 16S rRNA amplicon sequencing. Additionally, the survival, growth rate, age, size at the first reproduction, and neonate per female were assesed. The analysis revealed minor shifts in microbial composition following phage exposure. Nevertheless, results showed that the phage cocktail increased microbiome diversity. None of the life history parameters studied were affected by the presence of the phage cocktail, and no adverse effects were observed. The results indicated that under laboratory conditions the phage cocktail is safe for *D. magna* and its microbiome.

**Keywords:** *Daphnia magna*, Ecotoxicology, Phages, Host-associated microbiome, Life history parameters

**Supplementary materials:**

**
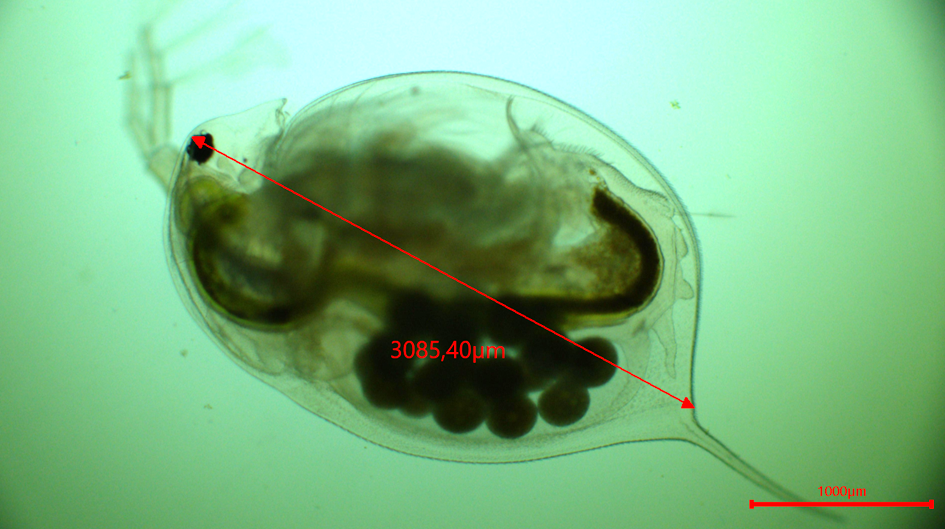
**

**Figure S1.** Representative microscopy image of *Daphnia magna* used for the measurement of body size at first reproduction (SFR). Body length was measured as the distance between the upper margin of the eye and the posterior edge of the carapace at the base of the tail spine, as indicated by the measurement line.

**Table S1.** Two-way ANOVA testing the effect of medium (i) ADaM – control, (ii) Phage treatment – four phages cocktail and genotype (B, D and P clone) of *D. magna* life history parameters: growth rate, age and size at the first reproduction and neonate number per female.

| Variable | Factor | df | F | p-value | N per group (within clone) |
| --- | --- | --- | --- | --- | --- |
| **Growth rate** |  |  |  |  |  |
|  | Medium | 1 | 0.76 | 0.3930 | 5 |
|  | Clone | 2 | 170.62 | **<0.001** |  |
|  | Medium x Clone | 2 | 1.76 | 0.1943 |  |
| Total 29 | Error | 24 |  |  |  |
| **Age at the first reproduction** | |  |  |  |  |
|  | Medium | 1 | 0.78 | 0.3799 | 9 – 10 |
|  | Clone | 2 | 32.12 | **<0.001** |  |
|  | Medium x Clone | 2 | 2.33 | 0.1072 |  |
| Total 58 | Error | 53 |  |  |  |
| **Size at the first reproduction** | |  |  |  |  |
|  | Medium | 1 | 0.12 | 0.7345 | 9 – 10 |
|  | Clone | 2 | 25.92 | **<0.001** |  |
|  | Medium x Clone | 2 | 1.35 | 0.2670 |  |
| Total 58 | Error | 53 |  |  |  |
|  |  |  |  |  |  |
| **Neonate number** |  |  |  |  |  |
|  | Medium | 1 | 3.81 | 0.564 | 8 – 10 |
|  | Clone | 2 | 36.11 | **<0.001** |  |
|  | Medium x Clone | 2 | 2.01 | 0.1446 |  |
| Total 56 | Error | 51 |  |  |  |

**
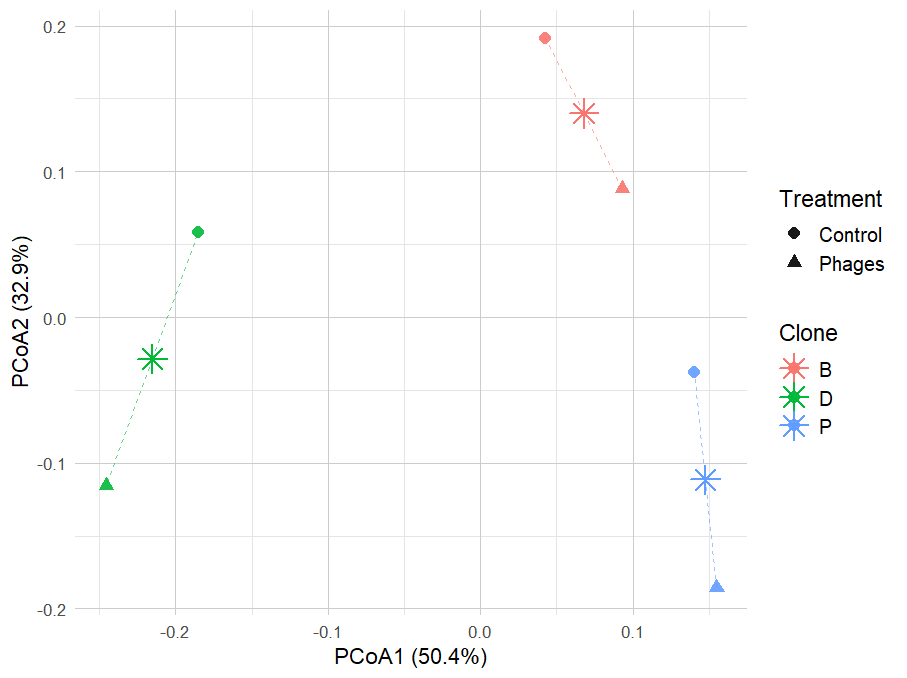
Figure S2.** Principal Coordinates Analysis (PCoA) of Bray–Curtis dissimilarities among bacterial communities from *D. magna* microbiome. Each point represents a biological replicate. Points are colored according to Clone and shaped according to Treatment (control vs. phage-exposed). Stars indicate clone centroids, and dashed lines connect each replicate to its clone centroid to illustrate within-clone dispersion. Ellipses were not drawn due to the low number of replicates per clone (n = 2). The plot visualizes patterns of beta diversity; statistical analyses of the effects of Clone and Treatment were performed using PERMANOVA (see Methods).

**
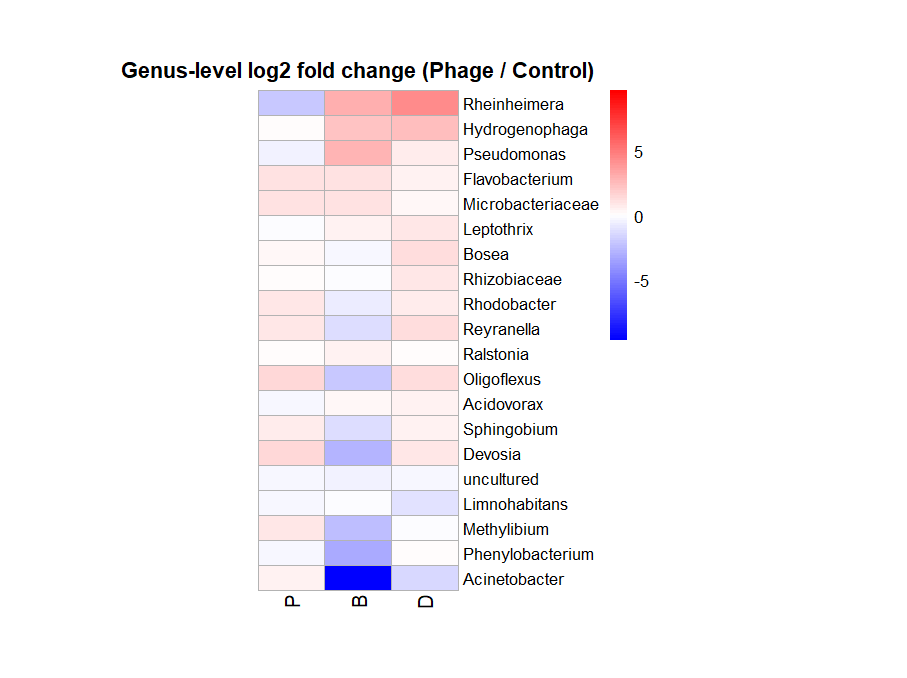
**

**Figure S3.** Genus-level log2 fold change in bacterial relative abundance in phage-exposed versus control samples across three *D. magna* clones (P, B, D). Heatmap colors represent log2 fold change, with red indicating an increase and blue indicating a decrease in phage-exposed samples relative to controls. Genera are sorted by the average log2 fold change across clones to highlight taxa with the strongest responses. No statistical tests are shown due to limited biological replicates (n = 3 clones); results are presented for exploratory visualization.
